# Effect of Hot Deformation on Phase Transformation Kinetics in Isothermally Annealed 3Mn-1.6Al Steel

**DOI:** 10.3390/ma13245817

**Published:** 2020-12-20

**Authors:** Adam Skowronek, Mateusz Morawiec, Aleksandra Kozłowska, Wojciech Pakieła

**Affiliations:** Department of Engineering Materials and Biomaterials, Silesian University of Technology, 18a Konarskiego Street, 44-100 Gliwice, Poland; mateusz.morawiec@polsl.pl (M.M.); aleksandra.kozlowska@polsl.pl (A.K.); wojciech.pakiela@polsl.pl (W.P.)

**Keywords:** medium-Mn steel, coiling simulation, dilatometry, retained austenite, inter-critical holding

## Abstract

The kinetics of ferritic transformation and the corresponding microstructural evolution in 0.17C-3.1Mn-1.6Al-0.04Nb-0.22Mo-0.22Si medium-Mn steel during isothermal annealing was investigated in dilatometric studies. The material was subjected to thermal and thermo-mechanical treatments aimed at obtaining, by the austenite → ferrite transformation, a sufficient fraction of ferrite to stabilize the retained austenite by C and eventual Mn partitioning. The samples were isothermally held for 5 h in a temperature range from 600 to 750 °C to simulate simplified temperature conditions of an industrial coiling process following hot rolling. Some of the samples were plastically deformed at a temperature of 900 °C before isothermal holding in order to study the effect of hot deformation on the kinetics of phase transformations. After the dilatometric investigations the material was subjected to light and scanning electron microscopy to reveal relationships between the holding temperature, deformation and microstructure evolution. Hardness tests were performed to assess the mechanical behavior. A significant effect of manganese in slowing down diffusional transformations during the cooling of steel was found. The influence of austenite deformation on the kinetics of austenite to ferrite transformation was noted. The plastically deformed samples showed an accelerated start of ferritic transformation and the extension of its range. During dilatometric tests, low-range dynamic ferritic transformation was recorded, which was also confirmed by the microscopic tests.

## 1. Introduction

Medium-Mn steels containing 3–12% Mn, which show the Transformation-Induced-Plasticity effect (TRIP), are of great interest to wide-world researchers [1,2,3]. Their advantage over conventional TRIP steels is the possibility of obtaining a retained austenite (RA) amount over 30% [4,5]. This metastable phase is responsible for high work hardening [6,7] of steel and thus the possibility of obtaining very high plasticity while maintaining good strength. Inter-critical annealing (IA) [8,9,10] is currently the main heat treatment process, which allows the obtaining of the highest fraction of retained austenite in medium-Mn steels. It is based on the austenite reverted transformation (ART) process [11,12], which allows the producing of lath-like duplex microstructures. It is used both for hot-rolled [13,14,15] and cold-rolled [16,17] sheet steels.

One of the few disadvantages of producing medium-Mn steels by IA is the need to reheat the material at least once (depending on a steel morphology [18,19]) after the hot/cold-rolling. For this reason, a method of producing these steel sheets based on thermo-mechanical processing is considered [20]. Apart from shortening the medium-Mn steel production by at least one step, which is associated with energy savings, it may result in additional grain refinement, which is common in this type of thermo-mechanical processes [20,21]. A typical thermo-mechanical processing of medium-Mn steels includes a bainitic step resulting in the producing of bainite-retained austenite mixtures. However, there are only a few studies [22,23] which focus on obtaining austenitic-ferritic microstructures in medium-Mn steels with this method. It requires an isothermal holding at the inter-critical region, instead of bainitic holding during cooling from finish rolling temperature. In this case, obtaining a stable retained austenite turned out to be difficult due to problems with slow kinetics of the ferrite formation from the initial austenite [24]. Moreover, from a thermo-mechanical point of view, it is possible to use the thermal energy of steel sheets during coiling following the hot rolling. This could be an energy-efficient spontaneous heat treatment taking the advantage of the inter-critical annealing region. At the starting point (after a few initial hours) of coiling, the temperature range of the steel sheets corresponds to the inter-critical annealing range.

Considering the above issues, this work is aimed at analyzing the kinetics of the ferritic transformation during the simplified physical coiling simulation of a lean medium-Mn steel. The interest is in checking if it is possible to stabilize some retained austenite is this way, typical for hot-rolled sheet products.

## 2. Materials and Methods

A chemical composition of the investigated steel, given in Table 1, was designed from the point of view of reducing Mn content in medium-Mn steels and limiting the precipitation of carbides (increased Al content with a limited Si content due to susceptibility to galvanizing [25]). Such a lean Mn content approach is especially important for maintaining economic steel production in the industry. The alloy was produced in a Balzers VSG-50 (Balzers, Balzers, Liechtenstein) vacuum furnace in the atmosphere of argon. 25 kg ingots were austenitized at 1200 °C for 3h and forged at a temperature range from 1200–900 °C to a thickness of 22 mm. The tests were performed in terms of dilatometry. Hence, the material was processed into cylindrical samples with a diameter of 4 mm and a length of 10 mm.

**Figure 1:**
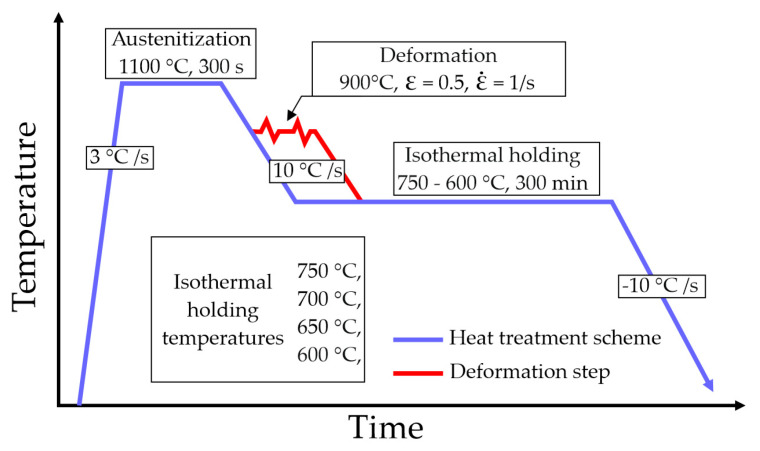
Schematic of heat treatments performed during the tests.

As part of the research, two methods of physical simulation of steel coiling were analyzed (Figure 1), which differ in the application or non-application of deformation. Such a division was used in order to determine the differences in the kinetics of phase transformations for the non-deformed and deformed austenite. In order to develop heat cycles, critical temperatures (Table 2) for the tested material were determined using dilatometry. The heat treatment cycle in both cases consisted of heating the sample at a rate of 3 °C/s to a temperature of 1100 °C for its austenitization. The holding time at the austenitizing temperature was 300 s. In the case of non-deformed samples the next step was cooling (at a rate of 10 °C/s) to isothermal holding temperature in a range from 750 to 600 °C, which corresponds to the typical temperatures of steel coiling following hot-rolling practices [26,27]. In case of deformed samples the next step was the deformation at 900 °C, to which the samples were cooled at a rate of −10 °C/s. The strain (ε) was 0.5 and the strain rate ($\dot{\varepsilon }$) was 1/s. After finishing the deformation, the samples were cooled to a selected annealing temperature corresponding to that of the undeformed samples (750, 700, 650 and 600 °C). In both cases the holding duration was 300 min (5 h). Due to the enormous thermal capacity of a multi-ton coil of sheet metal, even at high temperatures, it cools down very slowly, keeping the high temperature for many hours [27,28].

At the last step all samples were cooled to room temperature at a rate of 10 °C/s. The conducted tests have been significantly simplified in relation to industrial conditions due to their preliminary nature, in the case of medium manganese steels.

**Figure 2:**
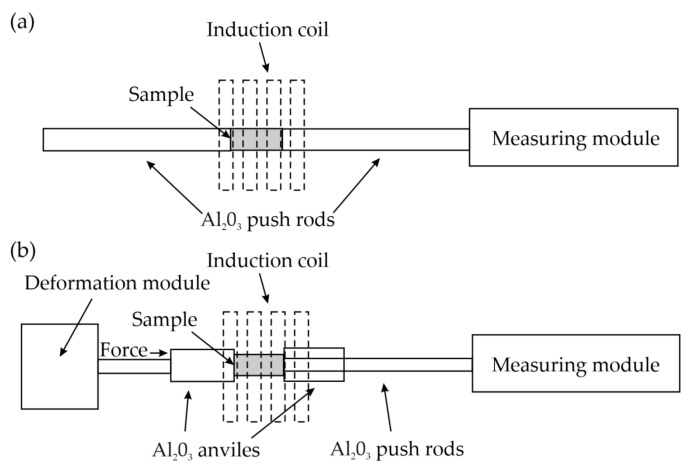
Schematic illustration of the used: (**a**) thermal and (**b**) deformation modules.

The heat- and thermo-mechanical treatment were performed using the BAHR dilatometer 805 A/D (BÄHR-Thermoanalyse GmbH, Hüllhorst, Germany) equipped with induction heating and a vacuum chamber. An illustration of the used deformation module is shown in Figure 2. The samples were cooled with argon. The temperature was recorded with a S-type thermocouple welded to the surface in the central part of the long side of the sample. This type of thermocouple mounting method gives information only on the temperature of the sample surface. The type of heating and cooling can create a temperature gradient in the sample that distorts the result in conventional steels. However, medium-manganese steels, as demonstrated in the study [7], due to the increased content of manganese strongly influencing the kinetics of the invasive transformation, are insensitive to slight differences in temperature and cooling rates.

During the investigations, the differences in sample length were recorded for further analyses of the kinetics of phase transformations. The dilatometric investigation were made according to ASTM A1033-04 Standard (ASTM International, Pennsylvania, USA) [29]. The analysis of dilatometric curves allows for a preliminary determination of the phases obtained in the steel [30]. However, further microstructural studies are necessary to confirm these results. For this purpose, light microscopic observations were first performed using the LSM Exciter 5 microscope (magnification 500×, Leica Microsystems, Wetzlar, Germany). A scanning electron microscopy (SEM) study using Zeiss SUPRA 35 (Carl Zeiss AG, Oberkochen, Germany) was performed for a more detailed analysis of the microstructure (magnification 10,000×). The metallographic specimens were prepared according to standard procedures taking into account the tendency to non-uniform deformation during uniaxial compression carried out in the dilatometer used. The non-deformed samples were cut perpendicular to a longitudinal direction in the central part. The deformed samples were cut longitudinally at a distance of 1/3 radius from the center, where the deformation obtained corresponds to the greatest extent to the given one. Microscopic observations included the central parts of the obtained samples (representing the microstructure and characterized by the highest measurement accuracy due to the proximity of the thermocouple). Etching was performed in nital. The ferrite fraction was calculated using ImageJ software. A hardness (and microhardness) test was carried out by the Vickers method using the Microhardness Tester FB-700 (Future-Tech Corp., Kawasaki-City, Japan) and a load of 10 N and 0.1 N.

## 3. Results

### 3.1. Non-Deformed Samples

**Figure 3:**
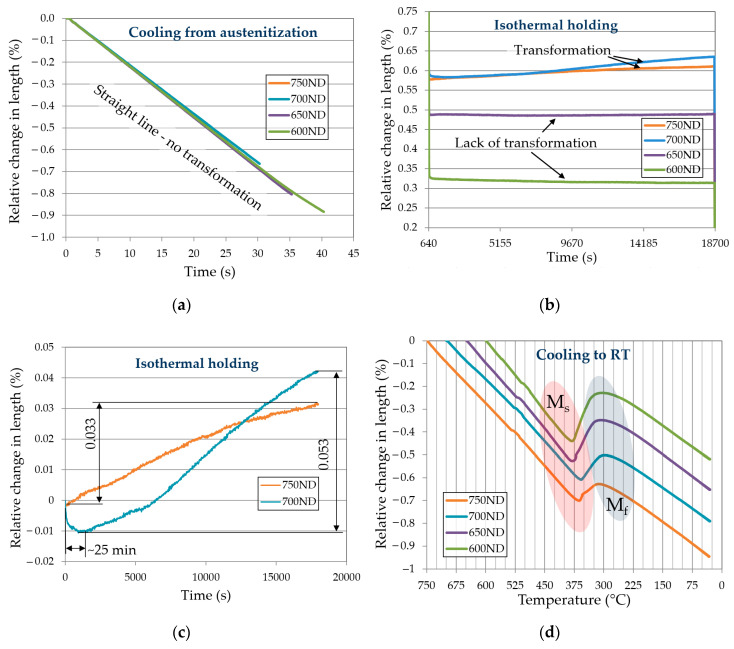
Dilatometric curves of non-deformed samples: (**a**) cooling from the austenitization to isothermal holding; (**b**) isothermal holding step for all non-deformed samples; (**c**) isothermal holding step for samples 700ND and 750ND—exhibiting the ferritic transformation; (**d**) cooling to room temperature showing a range of martensitic transformation; RT—room temperature.

The dilatometric curves (Figure 3) for all non-deformed (ND) samples exhibit no beginning of transformation during cooling to an isothermal step (Figure 3a, which took place after the end of austenitization. During the isothermal step, which is visible in Figure 3b, the samples 600ND and 650ND do not exhibit any transformation signals (the course is linear and flat). In case of samples 700ND and 750ND the relative change in length (RCL) increases with time. In this temperature range we see the beginning of the ferritic transformation, of which the kinetics is very slow.

The RCL of sample 700ND (Figure 3c) was 0.053%, which is over 60% more than in case of sample 750ND. This means a much higher (relatively, because both samples show very low RCL values) kinetics of the ferritic transformation at this temperature. The ferritic transformation of sample 700ND started with about a 25 min period of incubation. Figure 3d shows the dilatometric signal of martensitic transformation. The M_s_ (martensitic start) temperature values are collected in Table 3. The differences in the beginning of martensitic transformation between sample heat treated at different temperatures are not big. It is clearly visible that the samples 600ND and 650ND (in which there were any transformation during the isothermal holding) exhibit the highest M_s_ temperatures (388 °C and 385 °C). These results are the same as the bulk martensite start temperature determined by experimental method (Table 2). The samples 700ND and 750ND exhibit lower M_s_ temperatures (362 °C and 365 °C, respectively). The M_s_ values correspond to the RCL of samples during the isothermal step.

**Figure 4:**
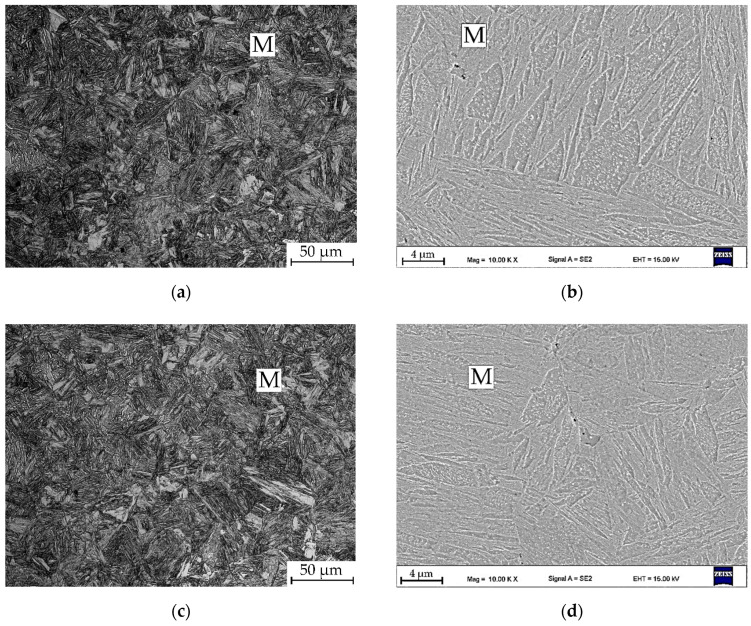

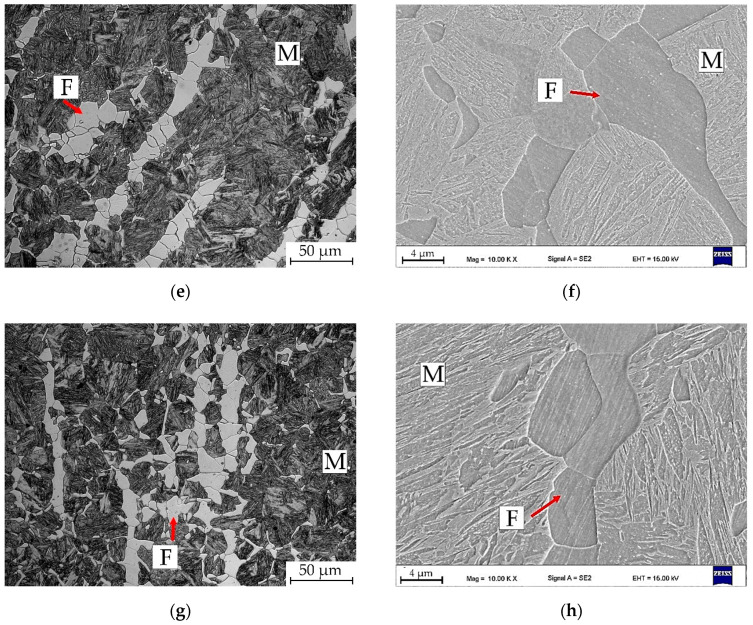
Light and SEM microscopic images of non-deformed samples: (**a**,**b**) sample 600ND; (**c**,**d**) sample 650ND; (**e**,**f**) sample 700ND; (**g**,**h**) sample 750ND; M—martensite; F—ferrite.

Figure 4 presents microscopic images of non-deformed samples. In case of samples 600ND (Figure 4a,b) and 650ND (Figure 4c,d) the microstructure consists of fine martensite, which is confirmed by hardness results (Table 3). The samples heat treated at higher temperatures (Figure 4e–h) contain also a fraction of equiaxial ferrite. The fraction of ferrite was calculated and is 16% for the sample 750ND and 12% for the sample 700ND (Table 3). The presence of ferrite has been confirmed by microhardness tests. Ferrite reduces the hardness (Table 3) of the alloy and is a reason for a big standard deviation in its value. The lowest hardness is exhibited by the 700ND sample, which contains the largest fraction of ferrite. The ferrite grains are arranged in bands, which is related to the segregation of manganese in medium-Mn steels, which was confirmed in earlier studies [31].

### 3.2. Deformed Samples

**Figure 5:**
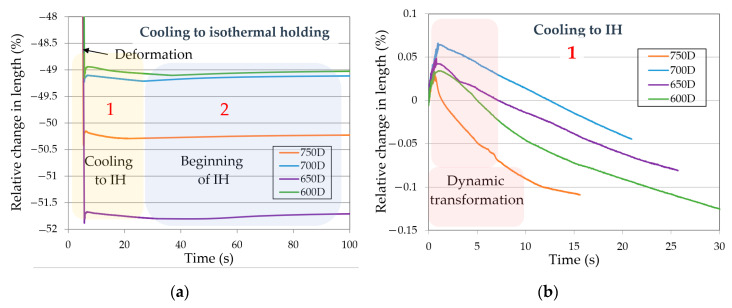

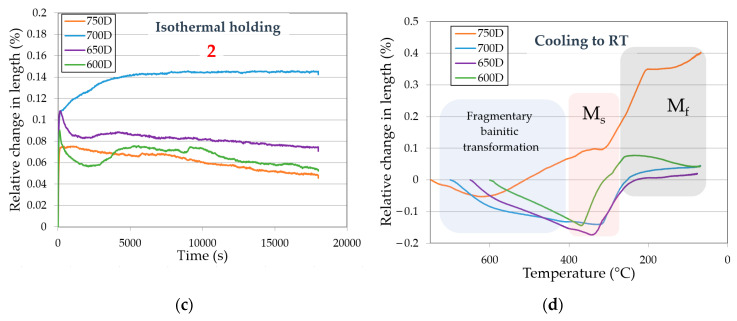
Dilatometric curves of deformed samples: (**a**) cooling from deformation to isothermal holding; (**b**) cooling from deformation to isothermal holding—magnification; (**c**) isothermal holding step; (**d**) cooling to room temperature; IH—isothermal holding; RT—room temperature.

The dilatometric curves of the deformed (D) samples are presented in Figure 5. Figure 5a presents a range of results from the ongoing deformation, through cooling to the holding temperature, and an initial fragment of the isothermal holding. It is visible that immediately after completing the deformation the RCL abruptly rises. The second increase is visible after the sample is cooled to the isothermal temperature. Both fragments are shown in Figure 5b,c, respectively. The RCL increase after the deformation in the most intense case is around 0.06%. The growth lasts for about 1.5s and clearly indicates a phase transformation. The conditions under which the transformation occurs indicate a dynamic ferritic transformation taking place during the plastic deformation below the A_C3_ temperature. This phenomenon is in line with previous studies [32,33,34]. Small differences in the course of the curves may be related to a band segregation of the alloying elements. Small, local differences in the chemical composition may affect the dynamics and the extent of the transformation, which is an argument supporting the thesis of dynamic transformation. After the first increase there is an RCL drop related to the samples’ cooling. After reaching the isothermal holding temperature the RCL again increases, which is related to the ferritic transformation start (or a continuation, taking into account the dynamic transformation). Samples held at different temperatures exhibit different curves during this stage.

The RCL of the sample 700D grows rapidly for the first 4000 s, reaching 0.145% and then stabilizes, indicating that the sample has reached a phase balance. In the case of the 650D and 600D samples, the RCL grows intensively during the first 100 s, reaching 0.11% and 0.09%, respectively. Next it drops by about 0.02% achieving a moderate stabilization. The sample 750D after an initial RCL increase of 0.075% begins to decrease its elongation, reaching 0.05% at the end of the isothermal holding. Therefore, the main contribution to the phase transformation of all samples is the initial increase, as the later variations in elongation are only minor. After the first 100–200 s of isothermal holding, there is mainly a redistribution of chemical elements. The RLC drops visible in case of the samples 650D and 600D may be related to presumable precipitation processes. During the final cooling stage there are two visible transformations. The first one, bainitic transformation, takes place in a region of higher temperature, around 600 °C. It is clearly visible in case of sample 750D, in which the RCL achieves ~0.15% (from isothermal holding to martensite formation start temperature). After the partial bainitic transformation the martensitic transformation starts, which is presented as a disturbance in the curve and its further rapid increase. According to the dilatometric signal the RCL during martensite formation was 0.25% (Figure 5d). This means that the structure should have more martensite then bainite in the final microstructure. As the isothermal temperature decreases, the transformation temperatures of bainite during cooling to RT decreases too. At 700 °C, the bainite transformation starts at around 550 °C and progresses to M_s_ temperature. However, looking at the signal from the dilatometer an amount of bainite should be much lower compared to the 750 °C isothermal holding. This trend with a decreasing bainite fraction is ongoing for lower isothermal holding temperatures. The M_s_ values are summarized in Table 4. Steels which have undergone the most extensive bainitic transformation present the lowest M_s_ temperatures, which is caused by the enrichment of the austenite in carbon and presumably in manganese.

**Figure 6:**
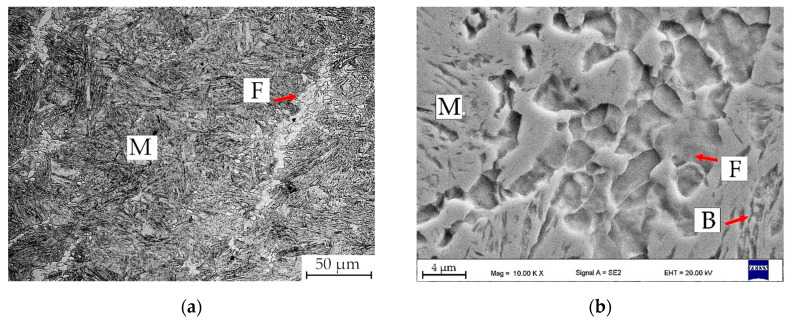

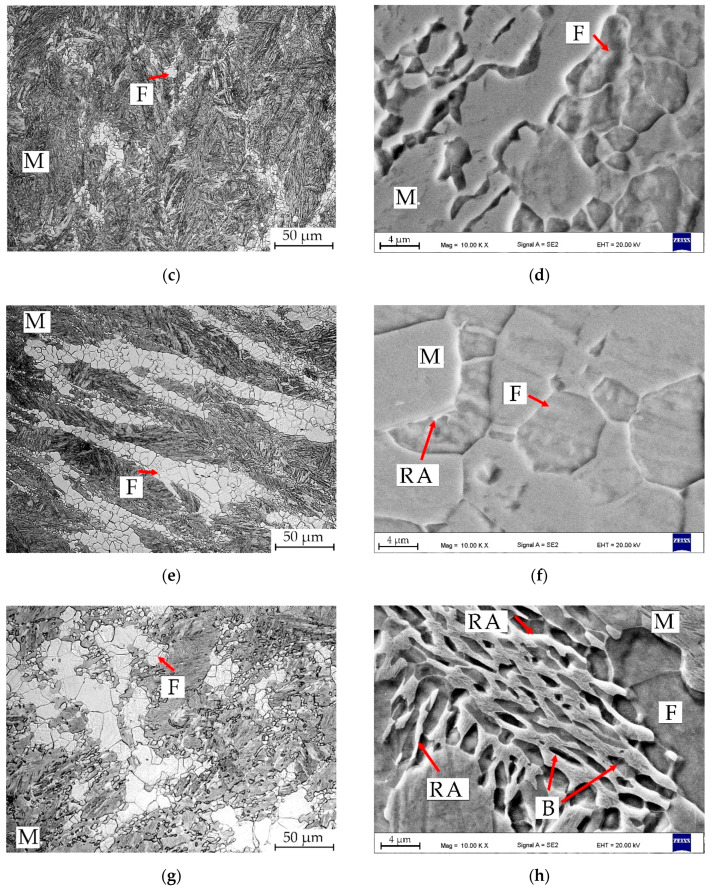
Light and SEM microscopic images of deformed samples: (**a**,**b**) sample 600D; (**c**,**d**) sample 650D; (**e**,**f**) sample 700D; (**g**,**h**) sample 750D; M—martensite; F—ferrite; B—bainite; RA—retained austenite.

The microstructures of deformed samples are presented in Figure 6. Generally, all samples exhibit the martensitic microstructure with various fractions of ferrite (from 24% to 4%) and bainite. What is interesting is that the deformed microstructures have two sizes of ferrite grains. Fine, globular ferrite grains are visible, which are not common in the case of the non-deformed samples. Such morphology may be related to the dynamic ferritic transformation and further recrystallization of this phase. Such results were earlier published and explained by Zhao et al. [33]. These characteristic necklace-like grains are formed on prior austenite grain boundaries.

The samples 600D and 650D present very similar microstructures containing martensite, ferrite and a small fraction of bainite. The size of ferrite deteriorates when decreasing the isothermal holding temperature, which is visible in Figure 6. These microstructures are in line with the dilatometric results. The sample 700D contains a higher fraction of bainite compared to the samples treated at lower temperatures. Only rarely visible are small grains of retained austenite located on martensite-ferrite boundaries. Austenite appears as white and smooth areas. Its morphology is elongated with sharp edges. It can be distinguished from the martensite as the latter shows a more “rough appearance” due to the fact that it is more prone to Nital etching [35]. The sample heat treated at 750 °C exhibits the higher (still small) fraction of retained austenite, which is located between the inter-lath ferrite/bainite grains. It has an elongated morphology increasing its stability in a mechanical way [36]. All microstructures contain small grains of fresh martensite, i.e., the thermally unstable austenite grains, which transformed into martensite in a final cooling stage to room temperature. The hardness results are presented in Table 4. The overall hardness results are lower compared to the non-deformed samples, which is related to the higher content of ferrite. The hardness drops with an increasing fraction of softer phases (ferrite and bainite).

## 4. Discussion

During the annealing of non-deformed samples, performed at 700 and 750 °C, ferritic transformation started but its extent was very sluggish, which resulted in obtaining a small amount of ferrite after 5 h. It should be noted that the ferritic alternation in conventional low carbon steels with Mn < 2 wt% is usually very fast. The increase in the Mn content by 1% significantly slowed the ferritic transformation. These results are confirmed by Hou et al. [22] and Ito et al. [23]. In addition, in the corresponding inter-critical annealing (ferrite (martensite) → austenite, instead of austenite → ferrite) the kinetics of the phase transformation is much faster, as evidenced by many publications, achieving a significant amount of ferrite in a short time [37,38].

The kinetics issues of phase transformations in Fe-C-Mn alloys are widely described [24,39,40]. Two limiting thermodynamic conditions were proposed [41] for the local interfacial equilibrium conditions: partitioned local equilibrium (PLE) which is controlled by manganese diffusion and negligible-partitioned local equilibrium (NPLE) controlled by carbon diffusion. Appropriate diagrams showing changes in the local interface equilibrium conditions as a function of the chemical composition of the Fe-C-Mn alloys can be found [24,41].

In most common medium-Mn steels the ferrite to austenite transformation starts under the negligible-partitioned local equilibrium (NPLE), which is controlled by fast carbon diffusion. As at this stage the ferritic matrix is only depleted in carbon, the state of the system moves towards the PLE mode. Wei et al. [42] simulated a transformation behavior of low-alloy steels. In the ferrite transformation, the NPLE/PLE transition takes place immediately after ferrite nucleation (<10^−3^ s) thus a large fraction of austenite grows under the PLE mode. In case of austenite → ferrite transformation, a ferrite growth starts under the PLA mode, which is controlled by sluggish manganese diffusion. During the process the composition of austenite shifts to higher carbon and manganese contents caused by increasing ferrite fraction. This causes the whole ferrite transformation to take place under the slow PLA mode.

The PLA mode dominates both in ferrite and austenite transformations but transformation rates are completely different. The ferrite (martensite) → austenite and austenite → ferrite transformations are controlled by manganese diffusion in the martensite and austenite matrix, respectively. Thus, the main factor influencing the kinetics of the above changes is the diffusion exponent which is ~100× higher in case of ferrite (martensite) according to MOB2 DICTRA database [24].

It is worth noting that such large differences in the kinetics of phase transitions do not occur in conventional steels containing, for example, 1.5% of manganese [43]. This is due to the fact that for steels containing up to 0.7% ofC, the transition from the NPE to PLE mode occurs only at the content of approx. 2% of manganese [24]. Thus, in these steels, phase transformations are controlled by the rapid diffusion of carbon.

**Figure 7:**
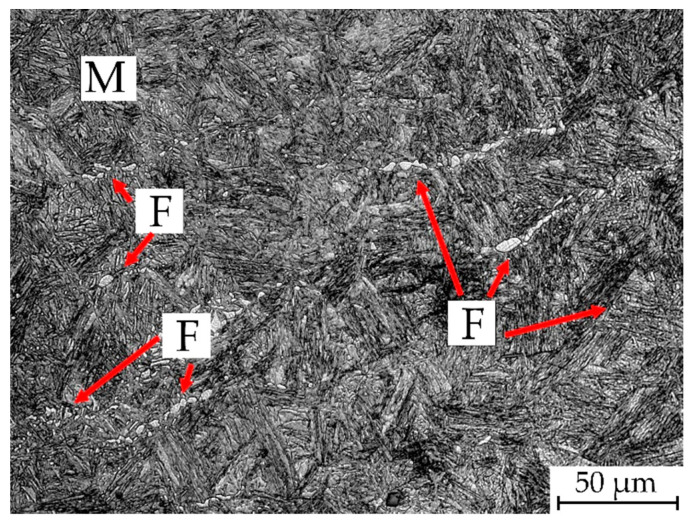
Microstructure of the sample quenched directly after deformation.

The deformed samples exhibit much higher kinetics of ferritic transformation (~0.1% in deformed samples to ~0.05% in non-deformed samples). The transformation also takes place during deformation, giving directly after it an increase of up to 0.06%. In order to determine the amount of dynamic ferrite, a sample was deformed at 900 °C and subsequently quenched at a cooling rate of 60 °C/s. The obtained microstructure is showed in Figure 7. Due to the high deformation temperature, the fraction of dynamic ferrite is 1.8 ± 0.3%, and its morphology is in line with the literature [23,34].

**Figure 8:**
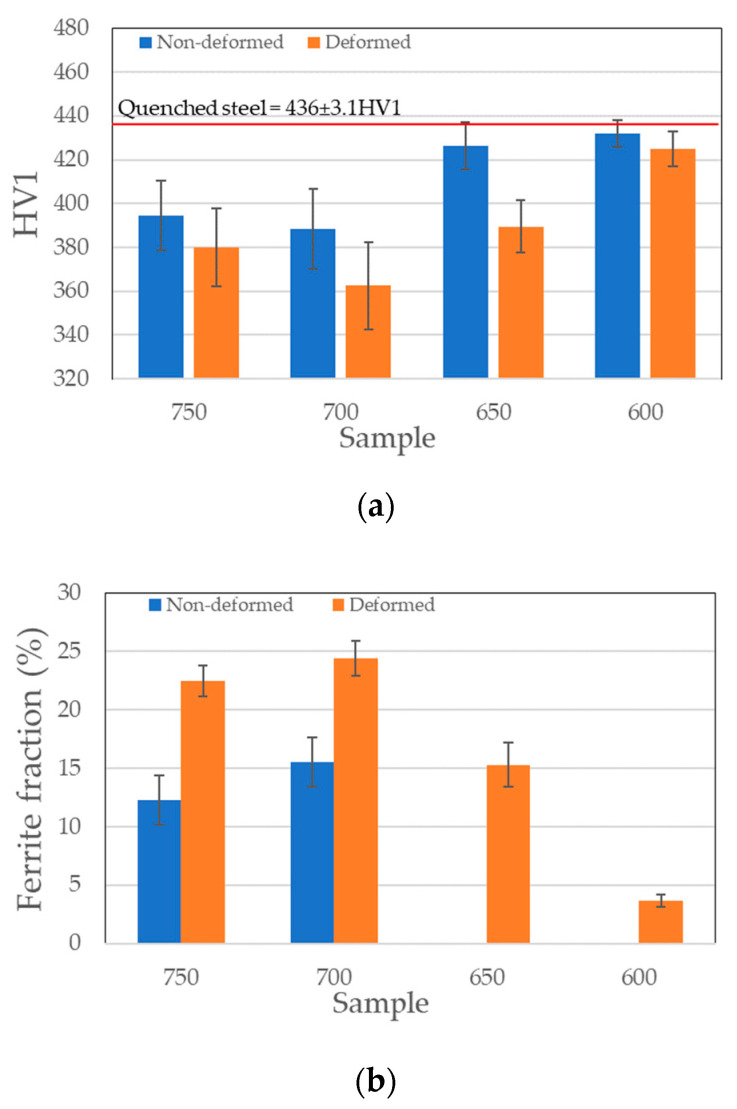
Comparison of: (**a**) hardness and (**b**) ferrite fraction for both groups of samples.

The start of transformation during isothermal holding is also significantly accelerated. In the case of undeformed samples 600ND and 650ND the transformation did not start for 5 h while after the deformation the transformation occurs immediately. Additionally, a small proportion of bainite in the samples was also obtained, which proves a significant “stimulation” of transformation by deformation. This phenomenon is caused by increase of the dislocation density in austenite during deformation. This leads to more preferential ferrite/bainite nucleation sites [12,13]. The hardness of the material (Figure 8a) changes inversely with the fraction of ferrite (Figure 8b). The highest fraction of ferrite and the lowest hardness in both types of treatments were obtained in samples isothermally treated at 700 °C.

**Figure 9:**
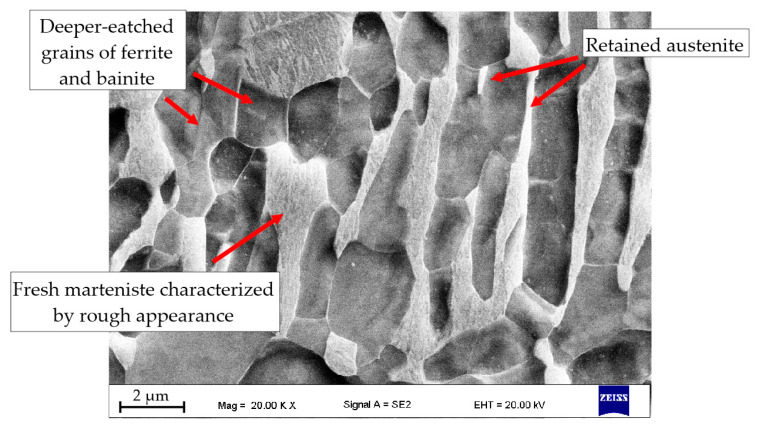
Characterization of the 750D sample morphology.

Despite the acceleration of the ferritic transformation by deformation, thus providing more time for the redistribution of the stabilizing elements, it was possible to obtain only a small amount of thermally stable retained austenite, which is visible in Figure 9. This phase is located between elongated ferrite grains, which ensure the best conditions for chemical and mechanical stabilizations. The proximity of a large fraction of ferrite and bainite allows these fragments of austenite to be enriched in carbon (and presumably by manganese), while a strongly elongated shape improves the mechanical stabilization [36]. The remaining part of austenite underwent a partial martensitic transformation due to worse conditions for stabilization. The result of the transformation is the presence of fresh martensite (Figure 9).

**Figure 10:**
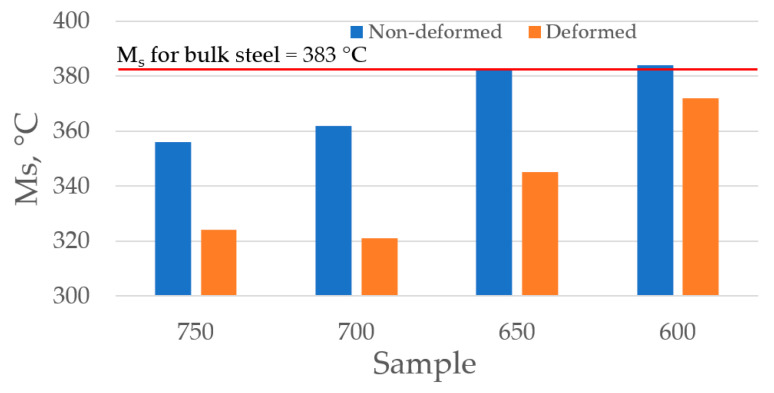
Comparison of M_s_ temperatures for both groups of samples.

The M_s_ comparison is presented in Figure 10. It is visible that the deformation allowed for reducing of the M_s_ temperature at each isothermal holding temperature. The highest reduction was obtained at 700 °C and is about 50 °C comparing to the non-deformed sample and over 60 °C comparing to the initial bulk material (100% martensite). Admittedly some reduction in M_s_ was achieved but sluggish ferritic transformation caused by the manganese transformation control and slow manganese diffusion make it impossible to obtain in this alloy a required ferritic-austenitic mixture. However, the performed research gives evidence for improvement of the process in alloys with a little higher amount of manganese combined with stronger deformation. This should accelerate the transformation kinetics and result in better stability of austenite at room temperature [44,45]. The works are in progress.

## 5. Conclusions

The work concerns a detailed study of the kinetics of the ferritic transformation during isothermal annealing following hot deformation of lean medium-Mn steel. The aim was to reduce the M_s_ temperature as low as possible and stabilize the retained austenite in a ferritic matrix of 0.17C-3.1Mn-1.6Al-0.04Nb-0.22Mo-0.22Si steel. The material has undergone heat treatment and thermo-mechanical treatment. The microstructure evolution was determined in terms of dilatometry and microscopic techniques. The main findings of the present study are as follows:

- The transformation of high-temperature austenite into ferrite is significantly hampered and delayed due to the control of this process by manganese diffusion. This makes it impossible to obtain an amount of ferrite suitable for the stabilization of the austenite by this method, even for long isothermal durations.
- The hot deformation at 900 °C resulted in a moderate amount of dynamic ferrite, especially beneficial at 750 and 700 °C.
- The application of deformation significantly accelerates and increases the kinetics of both ferritic and bainitic transformation of the tested steel. As a result, the higher ferrite fractions and resulting lower martensite start temperatures were noted for the plastically deformed samples.
- Despite the acceleration of the ferritic transformation and the 5h isothermal holding, it was possible to reduce M_s_ by only 60 °C and to obtain a small fraction of retained austenite in the steel held at 750 °C.
- The discussed method of heat treatment of medium manganese steels presents the possibility of obtaining austenitic-ferritic structures utilizing the industrial coiling practices. However, it requires the use of steels with a little bit higher manganese content and stronger accumulative deformations, which are closer to industrial conditions.

## Figures and Tables

**Table 1:** Chemical composition of the medium Mn steel used in the investigations.

Chemical Element	C	Mn	Al	Nb	Mo	Si
wt%	0.17	3.1	1.6	0.04	0.22	0.22

**Table 2:** Critical temperatures of the tested alloy.

Temperature, °C	A_c1_	A_c3_	M_s_
Dilatometric study	715	1013	389

**Table 3:** Experimental results of non-deformed samples.

Sample	M_s_, °C	Hardness			Ferrite Fraction, %
		HV1	HV0.01		
			Ferrite	Martensite	
600ND	388	432 ± 6	x	410 ± 4	x
650ND	385	426 ± 10	x	408 ± 3	x
700ND	362	388 ± 18	154 ± 6	404 ± 5	16 ± 1.8
750ND	356	394 ± 16	160 ± 4	406 ± 3	12 ± 2.1

**Table 4:** Experimental results of deformed samples.

Sample	M_s_, °C	Hardness			Ferrite Fraction, %
		HV1	HV0.01		
			Ferrite	Martensite	
600D	372	425 ± 8	170 ± 6	416 ± 5	4 ± 0.5
650D	345	389 ± 12	178 ± 7	413 ± 5	15 ± 1.9
700D	321	362 ± 20	172 ± 3	414 ± 3	24 ± 1.5
750D	324	380 ± 18	168 ± 4	412 ± 4	23 ± 1.3
